# Case report and literature review: key clinical management points and prognostic analysis of paraganglioma of the urinary bladder

**DOI:** 10.3389/fsurg.2026.1849321

**Published:** 2026-06-24

**Authors:** Chengbin Lu, Benmo Xu, Can Li, Sinan Yang, Xusong Pang, Weixiang Yang, Tianjian Xie, Zongyan Luo, Hanbin Li, Wei Luo, Zhiyu Shi, Libo Yang, Yong Yang

**Affiliations:** Department of Urology, The Third Affiliated Hospital of Kunming Medical University, Yunnan Cancer Hospital, Peking University Cancer Hospital Yunnan, Kunming, Yunnan, China

**Keywords:** bladder paraganglioma, catecholamine metabolites, perioperative management, pheochromocytoma, radical cystectomy

## Abstract

**Objective:**

To investigate the clinical characteristics, diagnostic approach, perioperative management, and key considerations in surgical decision-making for functional bladder paraganglioma.

**Methods:**

We conducted a retrospective analysis of the clinical data of a patient with pathologically confirmed bladder paraganglioma and discussed the findings in the context of relevant domestic and international literature.

**Results:**

The patient was a 50-year-old man who presented with frequent urination, urinary urgency, and dysuria for more than 2 months, accompanied by paroxysmal hypertension during urination with a peak blood pressure of 205/120 mmHg. Diagnostic transurethral resection of the bladder tumor was performed at another hospital, and postoperative pathology indicated paraganglioma. Upon admission to our hospital, further testing revealed markedly elevated plasma levels of norepinephrine and methoxy norepinephrine. Contrast-enhanced pelvic MRI revealed a mass on the right bladder wall involving the right ureteral orifice, accompanied by right-sided hydronephrosis and suspicious pelvic lymph nodes. After multidisciplinary discussion and thorough preoperative preparation including α-receptor blockade and volume expansion, the patient underwent radical cystectomy with bilateral pelvic lymphadenectomy and ileal neobladder reconstruction. Postoperative pathology confirmed bladder paraganglioma invading the full thickness of the bladder detrusor muscle (deep muscular layer) with a Ki-67 index of approximately 8%, and no lymph node metastasis was observed in 18 examined lymph nodes (7 left pelvic and 11 right pelvic). During the 5-month postoperative follow-up, no obvious recurrence or metastasis was detected.

**Conclusion:**

Urination-related paroxysmal hypertension is the pathognomonic sign of functional bladder paraganglioma. Preoperative catecholamine metabolite testing combined with pelvic contrast-enhanced MRI forms the core diagnostic system. Multidisciplinary collaboration-based perioperative management and individualized surgical resection are the keys to improving prognosis, and lifelong follow-up is mandatory for all patients.

## Introduction

Bladder paraganglioma (BPG) is particularly uncommon, accounting for only 0.3%–1.0% of all paragangliomas and 0.06%–0.09% of bladder tumors (1, 2). Clinical symptoms of BPG are nonspecific and often present as gross hematuria, urinary frequency, and urgency—typical signs of urinary tract irritation—making it easily confused with more common bladder tumors, such as bladder cancer and bladder leiomyoma. The preoperative misdiagnosis rate exceeds 60% (3). A 25-year systematic review conducted by Liu et al. demonstrated that early diagnosis could reduce the incidence of surgical complications from 35% to 10%, emphasizing the importance of accurate diagnosis and treatment (4). Currently, there is ongoing debate concerning the optimal surgical approach, the extent of lymph node dissection, pathological risk stratification, and long-term follow-up strategies for BPG (5). This article reports a case of functional bladder paraganglioma and, together with a review of the literature, summarizes essential points regarding diagnosis, perioperative management, and treatment decisions to enhance clinical recognition and standardize diagnosis and therapy.

## Case presentation

### General information

A male patient in his 50s was admitted to the hospital due to symptoms of frequent urination, urinary urgency, and dysuria that had persisted for more than 2 months without an identifiable cause. He did not report any accompanying symptoms, such as hematuria, abdominal pain, diarrhea, nausea, vomiting, chest tightness, or shortness of breath. The patient self-reported that he developed paroxysmal hypertension during urination, with a peak blood pressure of 205/120 mmHg, accompanied by transient headache and palpitations. The patient used a calibrated electronic sphygmomanometer to continuously monitor blood pressure at home for 1 week, recording 12 episodes of blood pressure surge during urination (systolic blood pressure 182–205 mmHg, diastolic blood pressure 105–120 mmHg), which spontaneously returned to baseline (125–135/75–85 mmHg) 10–15 min after urination. Three micturition provocation tests were performed during hospitalization in our hospital, all confirming that systolic blood pressure increased by ≥70 mmHg and diastolic blood pressure increased by ≥40 mmHg during urination, consistent with the patient's self-reported symptoms. During a follow-up visit at another hospital for kidney stones on March 19, 2025, a bladder tumor was discovered. The patient subsequently underwent a diagnostic transurethral resection of the bladder tumor. Postoperative pathology confirmed the tumor was infiltrating the lamina propria, consistent with a diagnosis of paraganglioma. He was then admitted to our hospital for further treatment. The patient had a 4-year history of hypertension, controlled with benazepril at a baseline level of approximately 130/81 mmHg. He had a history of left renal calculi surgery 3 years ago, a 20-year smoking history (quit 2 months ago), and no history of alcohol consumption or drug allergies.

### Tests

Preoperative endocrine and biochemical testing serves as the primary basis for diagnosing functional PPGL; the key results are summarized in Table 1.

**Table 1:** Key preoperative endocrine laboratory test results for the patient.

Test items	Test results	Reference range (standing)	Interpretation of results
Plasma norepinephrine (NE)	25,505.0 pmol/L	<10,054.0 pmol/L	Significantly elevated
Plasma epinephrine (E)	426.3 pmol/L	<545.8 pmol/L	Normal
Plasma dopamine (DA)	52.1 pmol/L	<196.0 pmol/L	Normal
Plasma methoxy norepinephrine (NMN)	12.21 nmol/L	<1.80 nmol/L	Six times the upper limit of normal; significantly elevated
Plasma methoxy epinephrine (MN)	0.62 nmol/L	<0.90 nmol/L	Normal
24-Hour urine methoxy norepinephrine (NMN)	722 nmol/24 h	<312 nmol/24 h	Significantly elevated
24-Hour urine methoxy epinephrine (MN)	186 nmol/24 h	<350 nmol/24 h	Normal

A PET/CT scan performed in March 2025 at the Department of Radiology of the external hospital demonstrated focal thickening of the right bladder wall with abnormal radiotracer uptake, consistent with paraganglioma. The lesion involved the right ureteral orifice, resulting in hydronephrosis and dilation of the right ureter. At the time of the scan, no significantly enlarged lymph nodes with increased metabolic activity were detected in the abdominal, pelvic, or retroperitoneal regions.

**Figure 1:**
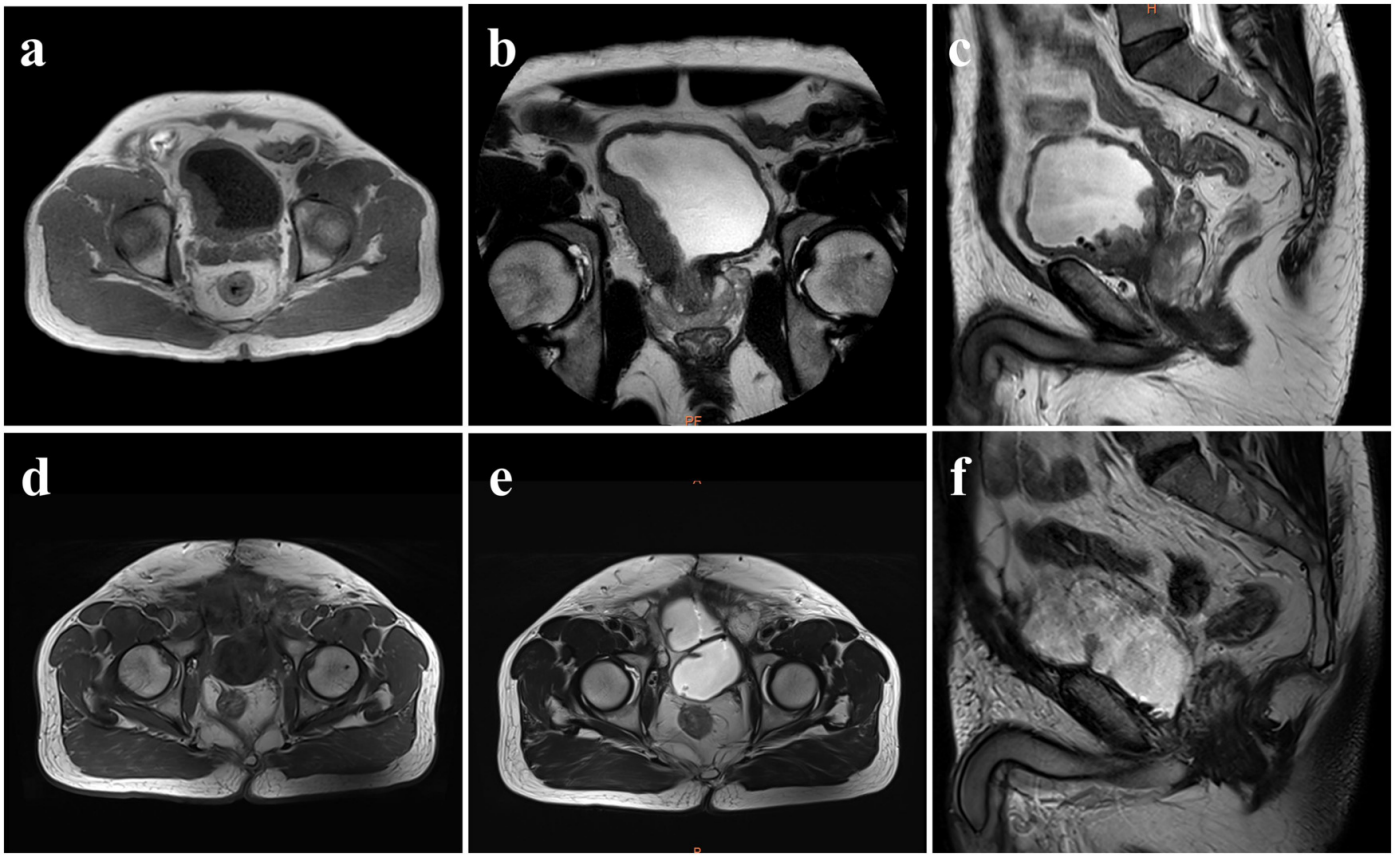
**(a–c)** Preoperative MRI image; **(d–f)** postoperative MRI image.

A contrast-enhanced pelvic MRI performed at our institution on March 28, 2025, identified a 2.2 cm mass on the right lateral wall of the bladder. On T2-weighted and diffusion-weighted imaging, the lesion exhibited a hyperintense signal and demonstrated significant enhancement following contrast administration. The mass is located near the right ureteral orifice and is associated with right-sided hydronephrosis. Imaging features indicated possible deep tumor infiltration with potential involvement of the perivesical fat, leading to a clinical staging assessment of T3N0M0. Several small lymph nodes were observed in the bilateral intra- and extra-iliac perivascular spaces and the presacral area, with the largest node measuring approximately 0.8 cm by 0.7 cm and some exhibiting heterogeneous enhancement. Regional lymph node metastasis could not be entirely excluded (refer to Figures 1a–c).

A contrast-enhanced pelvic MRI conducted at our hospital on August 6, 2025, revealed that “the bladder, prostate, and seminal vesicles had been resected, and an intestinal bladder substitute had been created in the pelvic cavity.” Additionally, a lymphatic cyst was observed near the left external iliac vessels, along with a small amount of fluid in the pelvic floor. No clear evidence of recurrence or metastasis was noted (see Figures 1d–f).

### Diagnosis and differential diagnosis

Based on the patient's significant elevation in blood pressure correlated with urination, marked abnormalities in catecholamine metabolites, prior pathological diagnosis of paraganglioma from another hospital, and MRI findings from our institution, a functional bladder paraganglioma was confirmed before surgery. Due to the deep infiltrative nature indicated by imaging, right-sided hydronephrosis, and suspicious regional lymph nodes, the clinical evaluation pointed to a locally advanced lesion with high metastatic potential. Bladder paraganglioma must be differentiated from common bladder tumors and benign lesions; the key differential points are summarized in Table 2.

**Table 2:** Differential diagnosis of bladder paraganglioma.

Disease type	Clinical presentation	Imaging findings	Pathological/Immunohistochemical features
Bladder paraganglioma	Urinary tract irritation + urination-related paroxysmal hypertension	T2WI hyperintense; arterial phase mild enhancement, venous phase sustained enhancement; no obvious extramural invasion in early stage	Cells arranged in nests; Syn (+), CgA (+), CK (−) (5)
Bladder cancer (urothelial carcinoma)	Predominantly painless gross hematuria	Cauliflower-like or papillary mass; marked arterial phase enhancement, rapid washout; prone to muscular layer invasion	Urothelial dysplasia; CK7 (+), CK20 (+) (6)
Bladder leiomyoma	Mostly asymptomatic; hematuria is occasionally observed	Well-defined hypoechoic nodule with homogeneous enhancement	Spindle cells arranged in bundles; SMA (+), Desmin (+), Syn (−) (7)
Benign prostatic hyperplasia with bleeding	Elderly males, primarily presenting with frequent urination and urinary urgency	Enlarged prostate; no clear tumor lesions within the bladder	Prostatic glandular epithelial hyperplasia: PSA may be mildly elevated

### Treatment

Prior to surgery, a multidisciplinary team (MDT) discussion was conducted, involving specialists from urology, anesthesiology, endocrinology (medical oncology), radiology, and pathology. The conclusions were as follows: (1) The patient was diagnosed with a functional bladder paraganglioma, with a high risk of severe hemodynamic fluctuations due to catecholamine release during surgery; (2) The tumor was located on the right bladder wall, involving the right ureteral orifice and associated with right-sided hydronephrosis. MRI findings suggested deep local invasion and suspicious regional lymph nodes; (3) To achieve complete tumor resection and obtain precise pathological staging, radical cystectomy with bilateral pelvic lymph node dissection was recommended. Preoperative preparation included adequate alpha-blocker therapy and volume expansion, with surgery planned once blood pressure was stabilized. Alpha-blockers (phenoxybenzamine) were administered for blood pressure control, along with volume expansion therapy for at least 7 days, aiming to achieve stable blood pressure, induce orthostatic hypotension, and allow moderate weight gain. The anesthesiology team prepared sodium nitroprusside, phentolamine and other antihypertensive agents, as well as norepinephrine, epinephrine and other vasopressors, to manage intraoperative hemodynamic fluctuations. In March 2025, the patient underwent radical cystectomy, prostatectomy with seminal vesiculectomy, bilateral pelvic lymphadenectomy, and ileal neobladder reconstruction under general anesthesia. During tumor dissection and mobilization of the lateral bladder ligaments, the patient experienced a sudden hypertensive episode, which was promptly managed with intravenous sodium nitroprusside by the anesthesiology team. After tumor excision, the patient developed refractory hypotension, requiring continuous norepinephrine infusion to maintain hemodynamic stability. Intraoperative blood loss was approximately 700 mL. Postoperatively, the patient was admitted to the intensive care unit (ICU) and later transferred to a general ward after vital signs stabilized.

**Figure 2:**
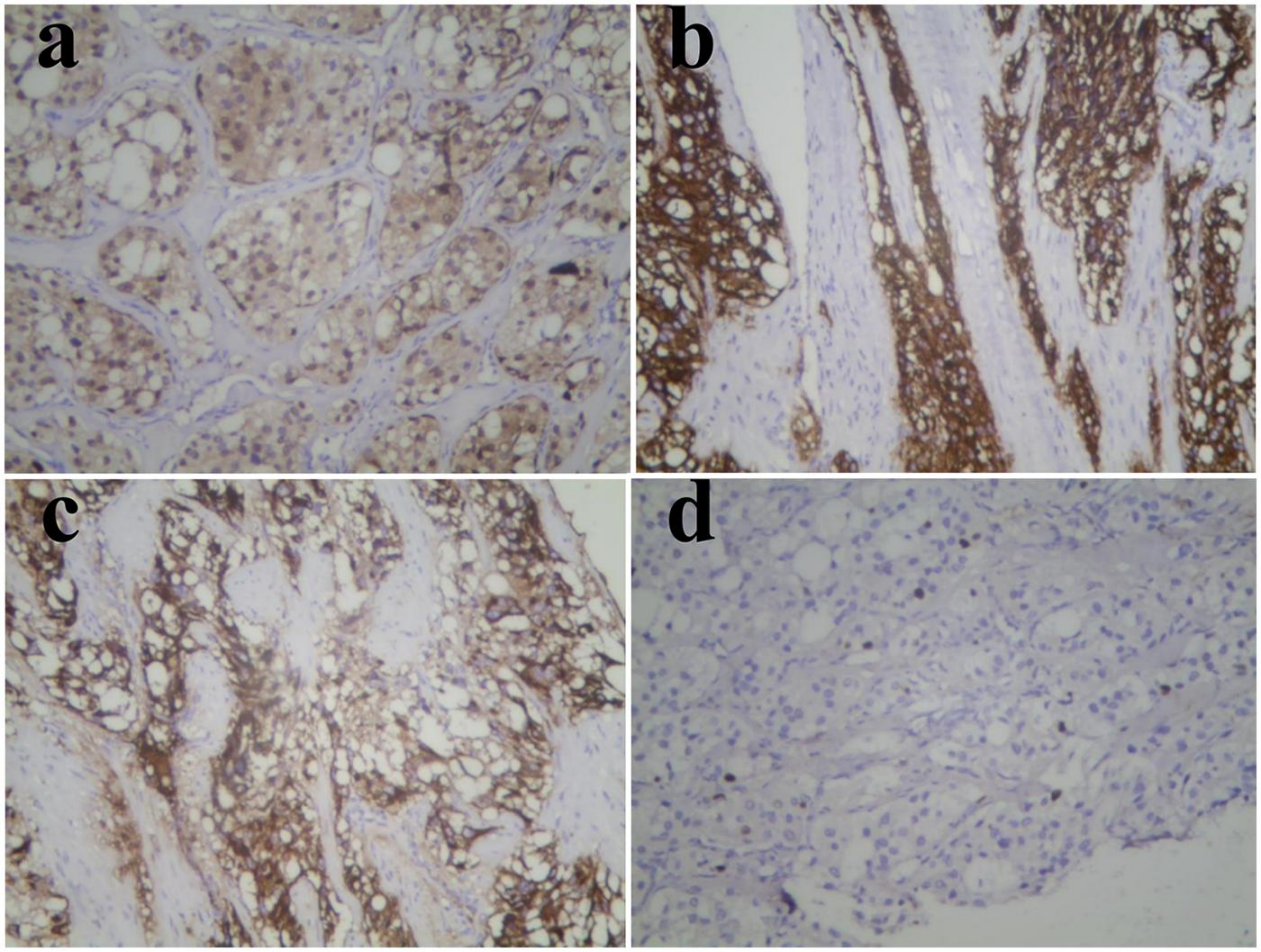
Immunohistochemical staining of bladder paraganglioma (×200). **(a)** Tumor cells show diffuse strong positive expression of Synaptophysin (Syn); **(b)** Tumor cells show diff use strong positive expression of Chromogranin A (CgA); **(c)** Tumor cells show moderate positive expression of CD56; **(d)** Sustentacular cells around tumor nests show scattered positive expression of S-100 protein. Brown staining indicates positive immunoreactivity; blue staining indicates hematoxylin-counterstained nuclei.

Postoperative gross specimen from this institution: The total cystectomy specimen revealed a tumor located on the right bladder wall. Postoperative microscopic and immunohistochemical findings from this institution: The tumor cells were arranged in nests (Zell Ballen pattern) and infiltrated the full thickness of the bladder detrusor muscle (deep muscular layer), without invasion of the perivesical fat tissue. Immunohistochemistry demonstrated Synaptophysin (Syn) positivity, Chromogranin A (CgA) positivity, S-100 positivity in supporting cells, CK negativity, GATA3 focal positivity, and a Ki-67 proliferation index of approximately 8%. Lymph node dissection results: None of the examined lymph nodes showed metastasis, including 0/7 left pelvic and 0/11 right pelvic nodes. Final pathological diagnosis: Bladder paraganglioma, invading the full thickness of the detrusor muscle, with medium-high metastatic potential (G1) according to the 2022 WHO classification of neuroendocrine tumors (8, 9) (see Figure 2). According to current classification criteria, an elevated Ki-67 index suggests a certain risk of biological aggressiveness, warranting long-term follow-up.

### Treatment outcomes, follow-up, and prognosis

The patient was transferred to the intensive care unit (ICU) for 24-hour postoperative monitoring. Blood pressure gradually stabilized following a continuous infusion of norepinephrine, and vasoactive agents were discontinued on the second postoperative day. The pelvic drainage tube was removed on postoperative day five, the ureteral stent on day twelve, and the urinary catheter on day fourteen. Urination was unobstructed, and no complications such as urinary leakage, hemorrhage, or infection occurred. The patient was hospitalized for a total of 16 days and was discharged without incident. At 5 months postoperatively, the patient returned for follow-up with a lower abdominal MRI. The imaging revealed: “The bladder, prostate, and seminal vesicles have been resected; an intestinal bladder substitute has been constructed in the pelvis, showing slightly thickened walls and contrast enhancement; a lymphatic cyst adjacent to the left external iliac vessels measuring approximately 1.6 cm × 1.4 cm, without enhancement; multiple small lymph nodes in both inguinal regions with a short axis <1.0 cm; minimal fluid collection in the pelvic floor. No definite evidence of recurrence or metastasis was observed.” The kidneys and retroperitoneum were unremarkable. According to the guidelines for the diagnosis and treatment of neuroendocrine tumors (10, 11), an individualized follow-up plan was implemented: ① Within 2 years postoperatively: Follow-up every 3 months with plasma and urinary methoxy adrenaline (MNs) levels and urinary tract ultrasonography; abdominal contrast-enhanced CT or MRI every 6 months to evaluate the pelvic and abdominal cavities. ② 2–5 years postoperatively: Biochemical and imaging examinations every 6 months, with annual whole-body bone scintigraphy or functional imaging (such as ^68^Ga-DOTATATE PET/CT) to screen for late metastasis. ③ Beyond 5 years: Annual follow-up is recommended and should be continued lifelong.

### Patient perspective

I had frequent and urgent urination for over 2 months, with a sharp rise in blood pressure when urinating that left me anxious. After being diagnosed with bladder paraganglioma, I feared surgical risks, but the medical team's detailed explanations and MDT personalized plan reassured me. The surgery went smoothly with meticulous care, and I recovered well post-operation, urinating normally now. Follow-ups show no abnormalities, and I'm truly grateful for the team's professional diagnosis, treatment and attentive care.

### Timeline of clinical events

**Figure 3:**
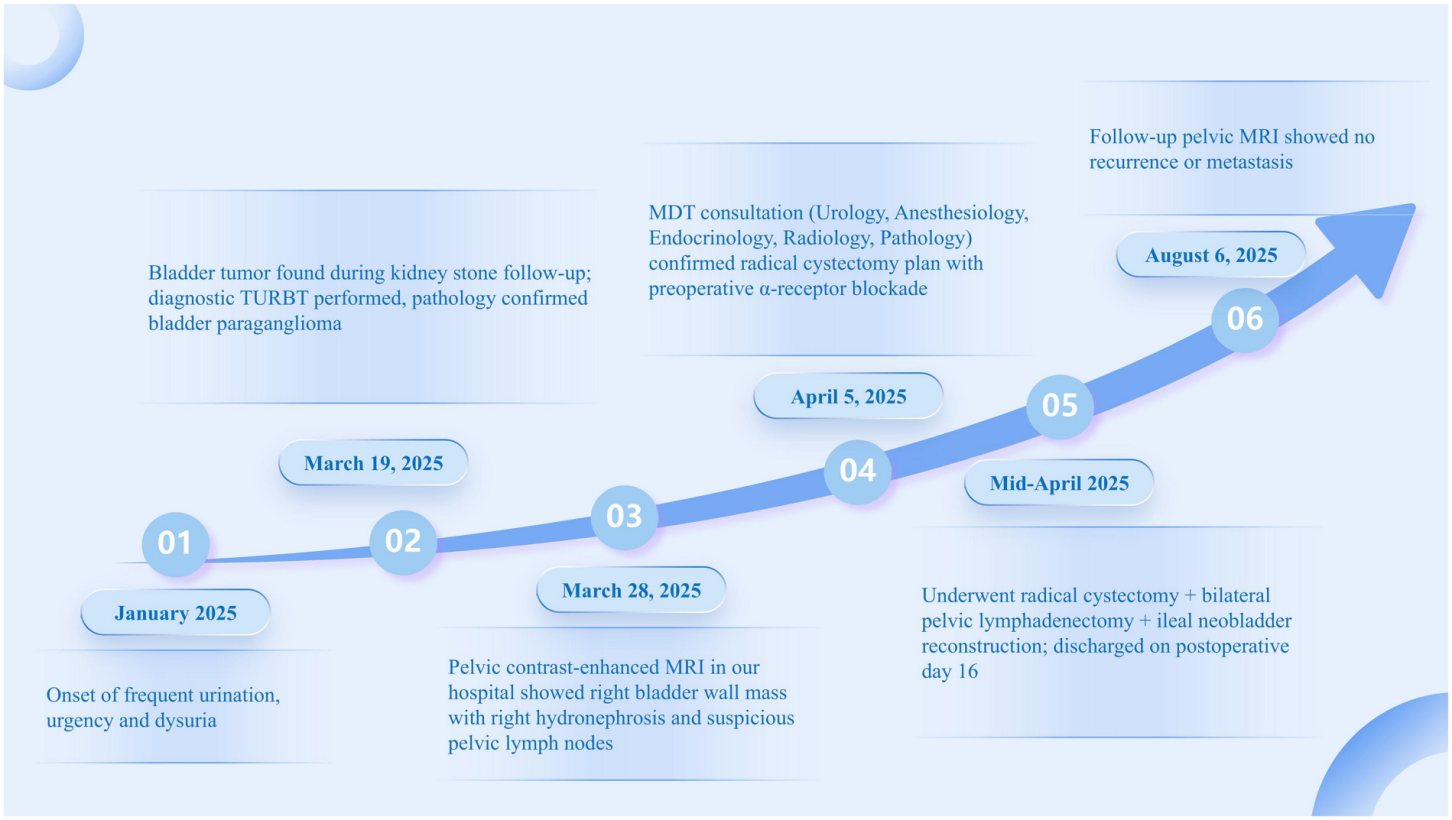
Summarizes the key milestones of the patient's care.

The key milestones of the patient's care are summarized in Figure 3.

## Discussion

The dramatic fluctuations in blood pressure during urination are the most characteristic feature of BPG. These fluctuations are caused by mechanical compression of the tumor during bladder filling and emptying, which leads to paroxysmal release of catecholamines into the bloodstream (12). This case highlights the importance of detailed medical history collection, especially the correlation between urination and blood pressure changes, which is the key to avoiding misdiagnosis. For patients with bladder masses accompanied by unexplained paroxysmal hypertension, timely testing of blood and urine catecholamine metabolites is essential (13).

Effective perioperative management is crucial for the success of these high-risk surgeries, and its foundation lies in the close collaboration of a multidisciplinary team (MDT). Proper preoperative pharmacological preparation and volume expansion are essential to stabilize circulation and prevent intraoperative hypertensive crises (14). Despite receiving adequate preoperative alpha-blocker therapy and volume expansion, the patient experienced a blood loss of 700 mL during the operation, and postoperative hypotension required the administration of vasoactive agents. This underscores the functional activity of these tumors and the associated perioperative risks (15). Consequently, meticulous intraoperative management, led by the anesthesiology team, is imperative. This includes invasive hemodynamic monitoring, goal-directed fluid therapy, and the availability of various vasoactive agents to manage the “roller-coaster” fluctuations in blood pressure (16). Postoperatively, transferring the patient to an intensive care unit (ICU) for continuous monitoring and support is a vital measure to ensure a smooth transition from vasoactive agent dependence and to prevent cardiovascular complications (17).

MRI is superior to ultrasound and CT in diagnosing BPG: T2-weighted imaging (T2WI) reveals slightly high signal intensity due to the abundance of neuroendocrine tumor cells and sparse cytoplasm, and contrast-enhanced scanning demonstrates “mild enhancement in the arterial phase and sustained enhancement in the venous phase,” distinguishing it from bladder cancer, which typically shows “marked enhancement in the arterial phase” (18). In this case, preoperative MRI suggested possible involvement of perivesical fat, leading to a clinical staging assessment of cT3NxM0 (locally advanced), which was the core basis for our decision to perform radical cystectomy. However, postoperative pathological examination confirmed that the tumor only invaded the full thickness of the detrusor muscle (deep muscular layer) without breaking through the muscularis propria to involve perivesical fat, with a final pathological stage of pT2bN0M0. This discrepancy between clinical imaging staging and pathological staging is a well-recognized clinical challenge, as MRI has a reported sensitivity of only 60%–70% in distinguishing between deep muscular invasion and microscopic perivesical fat involvement in bladder tumors. A whole-body bone scan and chest CT ruled out metastasis. The MRI features in this case—high signal on T2WI, persistent enhancement pattern, T3-stage invasion, and suspicious lymph nodes—were highly consistent with the pathophysiological characteristics of BPG, providing robust preoperative diagnostic evidence.

Treatment for BPG primarily involves surgical resection, with the principle being complete tumor removal while preserving bladder function. For T1–T2 tumors (confined to the bladder wall), laparoscopic partial cystectomy is the procedure of choice. Its advantages include minimal trauma (intraoperative blood loss <500 mL), rapid recovery (discharge 10–14 days postoperatively), and few complications (incontinence rate <5%) (19). The core difference between BPG and stage T2 bladder cancer lies in their fundamentally distinct biological behaviors, which directly lead to significant differences in treatment strategies and prognosis (20). As a medium-to-high-risk malignant tumor, stage T2 bladder cancer has a recurrence and metastasis rate of 30%–50% and is highly invasive (21, 22). The standard treatment regimen is radical cystectomy combined with standard pelvic lymph node dissection (23, 24). Some patients require adjuvant chemotherapy postoperatively; however, the treatment is highly invasive with a complication rate of 30%–40%, significantly impacting patients' quality of life due to urinary diversion (25). In contrast, bladder paraganglioma is a tumor with low malignant potential, exhibiting a metastasis rate of only 5%–8% and a recurrence rate of less than 5%. It shows no significant invasiveness. Even in stage T2 cases, partial cystectomy combined with selective lymph node dissection can achieve curative results. Postoperative radiotherapy or chemotherapy is not required, and the 5-year survival rate exceeds 90% (26).

**Figure 4:**
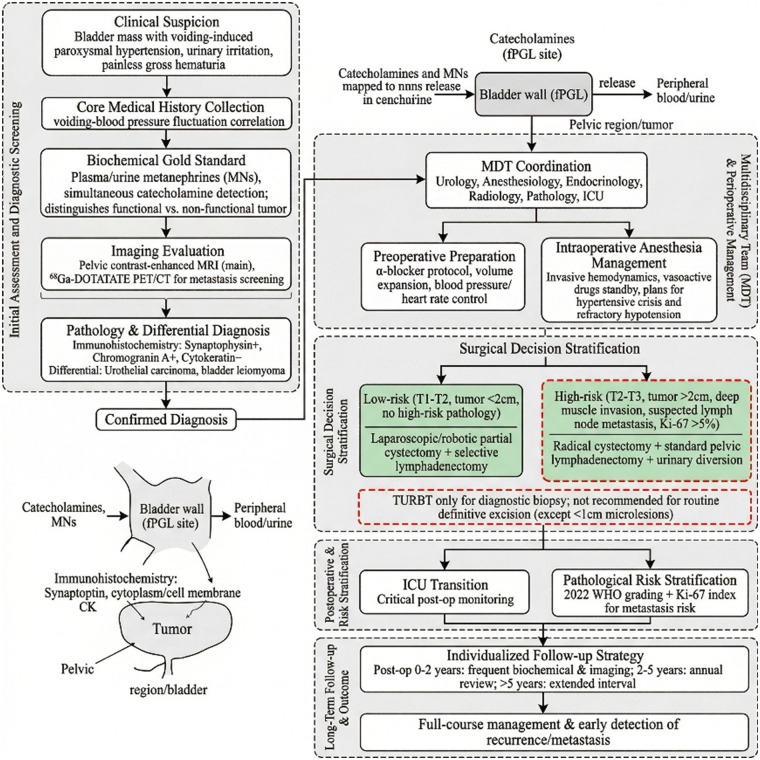
Standardized diagnostic and treatment decision-making flowchart for functional paragangliomas of the bladder.

In this case, radical cystectomy combined with prostatectomy and seminal vesiculectomy, bilateral pelvic lymph node dissection, and ileal neobladder reconstruction were performed for the following reasons: (1) The tumor was located at the right ureteral orifice, making partial cystectomy difficult to achieve negative margins; (2) Preoperative clinical staging was cT3NxM0 (locally advanced), and even the final pathological stage was pT2b (deep muscular invasion), which is associated with a significantly increased metastatic risk compared with T1 stage bladder paraganglioma; (3) The patient had undergone diagnostic TURBT at an external hospital, which carried a theoretical risk of tumor seeding along the resection tract; (4) The tumor diameter was 2.2 cm (>2 cm) and Ki-67 index was 8%, both of which are well-recognized high-risk factors for lymph node metastasis and late recurrence. Postoperative pathology confirmed negative lymph nodes in all 18 examined nodes, thereby providing the patient with a definitive curative result and accurate pathological staging. For T3–T4 tumors (involving extra-bladder tissue), radical cystectomy is required; however, such patients account for only 10% of BPG cases. The indications for pelvic lymph node dissection include preoperative imaging suggesting lymph node enlargement, tumor diameter >2 cm, or pathological evidence of high-risk features (Ki-67 > 5%) (27). In this case, the tumor diameter was 2.2 cm, and there were suspicious lymph nodes on imaging, so bilateral pelvic lymph node dissection was performed. Postoperative pathology revealed no metastasis, thereby confirming the staging and preventing a missed diagnosis. Based on the experience of this case and a comprehensive review of the literature, we have constructed a standardized diagnostic and treatment decision-making flowchart for functional bladder paraganglioma (Figure 4).

This study has several important limitations that should be acknowledged. First, this is a single-case report with a small sample size, and the conclusions may not be generalizable to all patients with bladder paraganglioma, especially those with non-functional tumors or metastatic disease. Second, the follow-up period is only 5 months, which is insufficient to evaluate long-term recurrence and metastasis risks, particularly for tumors with medium-high metastatic potential such as this case (pT2b, Ki-67 8%). Third, genetic analysis was not performed on the tumor tissue or the patient's peripheral blood. Approximately 30%–40% of paragangliomas are associated with germline mutations in genes such as SDHB, SDHD, and RET, and genetic testing is critical for assessing familial cancer risk, guiding personalized follow-up strategies, and identifying potential targeted therapy options. Future research should include larger multicenter cohorts and longer follow-up periods to further refine the risk stratification system and optimize the diagnosis and treatment protocols for bladder paraganglioma.

## Conclusion

Urination-related paroxysmal hypertension is the pathognomonic sign of functional bladder paraganglioma. Preoperative catecholamine metabolite testing combined with pelvic contrast-enhanced MRI forms the core diagnostic system. Multidisciplinary collaboration-based perioperative management and individualized surgical resection are the keys to improving prognosis, and lifelong follow-up is mandatory for all patients.

## Data Availability

The original contributions presented in the study are included in the article/Supplementary Material, further inquiries can be directed to the corresponding authors.

## References

[1] Matsumoto S Ishikawa Y Fukushima H Yamamoto K Tsujimoto K Kimura K. A case of bladder paraganglioma completely resected by transurethral endoscopic en-bloc resection of bladder tumor. IJU Case Rep. (2025) 8:93–6. 10.1002/iju5.12811 40034905 PMC11872215

[2] Shekhda KM Palan JM Albor CB Wan S Chung T-T. A rare case of bladder paraganglioma treated successfully with robotic partial cystectomy. Endocr Oncol. (2024) 5:e240044. 10.1530/EO-24-0044 PMC11728871 39810845

[3] Aryal S Sharma S Shrestha S Parajuli S Duwadee P Bista P. A rare case report of urinary bladder paraganglioma initially misdiagnosed as a psychiatric disorder. Ann Med Surg. (2025) 87:4651–4. 10.1097/MS9.0000000000003429 PMC12369839 40851990

[4] Liu Q Wang T Tu W Zhou P Wu X Lv H. Bladder paraganglioma: a 25-year systematic review unveils the benefits of early diagnosis in reducing surgical complications. Front Surg. (2025) 12:1657833. 10.3389/fsurg.2025.1657833 41059261 PMC12497724

[5] Calleja Duran I Hernandez Sanchez JE Popescu OB. An unusual entity in urology: urinary bladder paraganglioma. J Med Cases. (2025) 16:287–92. 10.14740/jmc5145 40904758 PMC12404112

[6] Li H Xie J Chen Z Yang S Lai Y. Diagnosis and treatment of a rare tumor-bladder paraganglioma. Mol Clin Oncol. (2020) 13:40. 10.3892/mco.2020.2110 32832083 PMC7439150

[7] Co JL Goco MLL So JS. Paraganglioma in the urinary bladder: a pitfall in histopathologic diagnosis. Acta Med Indones. (2023) 55:95–100. 36999255

[8] Mete O Asa SL Gill AJ Kimura N De Krijger RR Tischler A. Overview of the 2022 WHO classification of paragangliomas and pheochromocytomas. Endocr Pathol. (2022) 33:90–114. 10.1007/s12022-022-09704-6 35285002

[9] Rindi G Mete O Uccella S Basturk O La Rosa S Brosens LAA. Overview of the 2022 WHO classification of neuroendocrine neoplasms. Endocr Pathol. (2022) 33:115–54. 10.1007/s12022-022-09708-2 35294740

[10] Shah MH Goldner WS Benson AB Bergsland E Blaszkowsky LS Brock P. Neuroendocrine and adrenal tumors, version 2.2021, NCCN clinical practice guidelines in oncology. J Natl Compr Canc Netw. (2021) 19:839–68. 10.6004/jnccn.2021.0032 34340212

[11] Garcia-Carbonero R Matute Teresa F Mercader-Cidoncha E Mitjavila-Casanovas M Robledo M Tena I. Multidisciplinary practice guidelines for the diagnosis, genetic counseling and treatment of pheochromocytomas and paragangliomas. Clin Transl Oncol. (2021) 23:1995–2019. 10.1007/s12094-021-02622-9 33959901 PMC8390422

[12] Degrieck B De Visschere P Lapauw B. Bladder paraganglioma. J Belg Soc Radiol. (2020) 104:25. 10.5334/jbsr.2064 32435750 PMC7227393

[13] Withey SJ Christodoulou D Prezzi D Rottenberg G Sit C Ul-Hassan F. Bladder paragangliomas: a pictorial review. Abdom Radiol. (2022) 47:1414–24. 10.1007/s00261-022-03443-2 35157102

[14] Bohensky J Daher N. Hypertensive crisis. J Am Acad Physician Assist. (2024) 37:45–6. 10.1097/01.JAA.0000000000000026 38985116

[15] Mehmood Y Gupta R Mahajan A Rahman Y Gupta K. Incidental paraganglioma of the urinary bladder: a case series highlighting a rare entity and a urologist’s nightmare. Bladder. (2025) 12:e21200052. 10.14440/bladder.2024.0064 40933481 PMC12417874

[16] Stewart MH. Hypertensive crisis: diagnosis, presentation, and treatment. Curr Opin Cardiol. (2023) 38:311–7. 10.1097/HCO.0000000000001049 37016936

[17] Ibrahim J Jacobsen TP Boulos A DeFrancisis JS Simhadri P. Managing a patient with hypertensive crisis. Cureus. (2025) 17:e90003. 10.7759/cureus.90003 40951055 PMC12431168

[18] Zhang J Bai X Yuan J Zhang X Xu W Ye H. Bladder paraganglioma: CT and MR imaging characteristics in 16 patients. Radiol Oncol. (2021) 56:46–53. 10.2478/raon-2021-0055 34973050 PMC8884856

[19] Orsini A Ferretti S Tamborino F Cicchetti R Ciavarella D Seccia B. Mastering bladder paraganglioma for optimal treatment: a case report of robot-assisted surgery. Ther Adv Urol. (2024) 16:17562872241249603. 10.1177/17562872241249603 38779495 PMC11110518

[20] Liang J Yin L Gao L Yin L Ren W Jin Z. Contrast-enhanced CT in the differential diagnosis of bladder cancer and paraganglioma. Abdom Radiol. (2024) 49:1584–92. 10.1007/s00261-024-04217-8 38502213

[21] Folgosa Cooley L Weiner AB Meng X Woldu SL Meeks JJ Lotan Y. Survival by T stage for patients with localized bladder cancer: implications for future screening trials. Bladder Cancer. (2021) 7:23–31. 10.3233/BLC-200381 38993212 PMC11181799

[22] Zhao J Li J Zhang R. Off the fog to find the optimal choice: research advances in biomarkers for early diagnosis and recurrence monitoring of bladder cancer. Biochim Biophys Acta Rev Cancer. (2023) 1878:188926. 10.1016/j.bbcan.2023.188926 37230421

[23] Hausmann J Grunewald CM. Kann beim muskelinvasiven urothelkarzinom der harnblase zukünftig auf die zystektomie verzichtet werden?: Neue daten zur trimodalen therapie und zum blasenerhalt nach reiner systemtherapie. Urologie. (2024) 63:985–93. 10.1007/s00120-024-02420-5 39143395

[24] Huelster HL Mason NT Davaro F Naqvi SMH Kim Y Gilbert SM. Cost-utility of initial management of high-grade T1 bladder cancer with intravesical BCG vs immediate radical cystectomy. Urology. (2024) 187:106–13. 10.1016/j.urology.2024.02.033 38467285

[25] Wang A Chen M Li D Shi J Tang W Zhang Z. Disitamab vedotin alone or in combination with immune checkpoint inhibitors in bladder-sparing treatment of muscle-invasive bladder cancer: a real-world study. Clin Genitourin Cancer. (2024) 22:102085. 10.1016/j.clgc.2024.102085 38636170

[26] Qin J Zhou G Chen X. Imaging manifestations of bladder paraganglioma. Ann Palliat Med. (2020) 9:346–51. 10.21037/apm.2020.03.09 32233638

[27] Treccani LP Artoni F Brancelli C Veccia A D’Onofrio M Pichiri I. Case of the month’ from the university of Verona, Italy—navigating the medical and surgical challenges of urinary bladder paraganglioma: insights from a clinical case. BJU Int. (2025) 135:743–7. 10.1111/bju.16596 39548949 PMC11975215

